# Analysis of microRNA Expression Profiles Induced by Yiqifumai Injection in Rats with Chronic Heart Failure

**DOI:** 10.3389/fphys.2018.00048

**Published:** 2018-02-06

**Authors:** Yu Zhao, Yunfei Li, Ling Tong, Xinying Liang, Han Zhang, Lan Li, Guanwei Fan, Yi Wang

**Affiliations:** ^1^Pharmaceutical Informatics Institute, College of Pharmaceutical Sciences, Zhejiang University, Hangzhou, China; ^2^State Key Laboratory of Core Technology in Innovative Chinese Medicine, Tasly Academy, Tasly Holding Group Co., Ltd., Tianjin, China; ^3^Tianjin State Key Laboratory of Modern Chinese Medicine, Tianjin University of Traditional Chinese Medicine, Tianjin, China

**Keywords:** Yiqifumai injection, miRNA expression profiling, chronic heart failure, traditional Chinese medicine, hypertrophy, apoptosis

## Abstract

**Background:** Yiqifumai Injection (YQFM) is clinically used to treat various cardiovascular diseases including chronic heart failure (CHF). The efficacy of YQFM for treating heart failure has been suggested, but the mechanism of action for pharmacological effects of YQFM is unclear.

**Methods:** Echocardiography detection, left ventricular intubation evaluation, histopathology and immunohistochemical examination were performed in CHF rats to evaluate the cardioprotective effect of YQFM. Rat miRNA microarray and bioinformatics analysis were employed to investigate the differentially expressed microRNAs. *In vitro* models of AngII-induced hypertrophy and t-BHP induced oxidative stress in H9c2 myocardial cells were used to validate the anti-hypertrophy and anti-apoptosis effects of YQFM. Measurement of cell surface area, ATP content and cell viability, Real-time PCR and Western blot were performed.

**Results:** YQFM significantly improved the cardiac function of CHF rats by increasing left ventricular ejection fraction and fractional shortening, decreasing left ventricular internal diameter and enhancing cardiac output. Seven microRNAs which have a reversible regulation by YQFM treatment were found. Among them, miR-21-3p and miR-542-3p are related to myocardial hypertrophy and cell proliferation, respectively and were further verified by RT-PCR. Target gene network was established and potential related signaling pathways were predicted. YQFM could significantly alleviate AngII induced hypertrophy in cellular model. It also significantly increased cell viabilities and ATP content in t-BHP induced apoptotic cell model. Western blot analysis showed that YQFM could increase the phosphorylation of Akt.

**Conclusion:** Our findings provided scientific evidence to uncover the mechanism of action of YQFM on miRNAs regulation against CHF by miRNA expression profile technology. The results indicated that YQFM has a potential effect on alleviate cardiac hypertrophy and apoptosis in chronic heart failure.

## Introduction

Chronic heart failure (CHF) refers to a progressive syndrome of heart failure with high morbidity and mortality (Ramani et al., 2010; Segers and De Keulenaer, 2013). Clinical observations show that several syndromes have high risk inducing CHF, such as hypertension (He et al., 2001), age factors (Fukuta and Little, 2007) and neurohormonal system disorder (Landmesser et al., 2009), etc. Common used therapies for CHF include angiotensin converting enzyme inhibitors (Berliner and Bauersachs, 2017), beta-receptor blockers (Oniorisan and Lanfear, 2014), mineralocorticoid receptor antagonist (Liu et al., 2015), diuretics (Faris et al., 2012) and other modern medicines.

Compared to the conventional treatment by small molecule drugs, Traditional Chinese Medicine (TCM) including botanical drug, provides a more flexible approach for individualized medicine with better efficacy and less side effects (Xu, 2009). Shengmaifang, a particular prescription of TCM, has obvious clinical curative effects on preventing or curing CHF (Fu et al., 2010). Yiqifumai Injection (YQFM), derived from Shengmaifang, is composed of *Panax ginseng, Ophiopogon japonicas*, and *Schisandra chinensis.ginseng* (Li et al., 2015). It is a modern botanical drug to treat microcirculatory disturbance-related diseases (Yuan et al., 2011). Several studies showed that YQFM can exert cardioprotective effects through protecting cardiomyocytes, preventing myocardial calcium overload (Yang et al., 2017), exhibiting anti-hypoxic function (Feng et al., 2016), apoptosis reduction (Li et al., 2016), anti-inflammatory (Xing et al., 2013) and other pharmacological regulations. However, investigation on pharmacological mechanism of YQFM for treating CHF has been hampered due to the lacking of holistic strategies.

MicroRNAs (miRNAs) are noncoding regulatory single stranded small RNAs with short length (19~25 nucleotides in general) (Kuo and Ying, 2013). miRNAs play pivotal roles in regulating cell proliferation, differentiation and apoptosis by degrading down street mRNAs or inhibiting protein expression (Bartel, 2009; Kuo and Ying, 2013; Ciszek et al., 2015). Aberrant miRNA profiles have been reported to associate with several diseases such as myocardial diseases and cancers in human (Hayes et al., 2014; Olson, 2014). For miRNA profiling study, microarray technique makes it possible to detect hundreds of miRNAs expression efficiently (Wu et al., 2013; Rong et al., 2015). The technique mainly focuses on mining and identifying new or already known miRNAs among abundance of differentially expressed miRNAs (Rong et al., 2015). By further predicting target genes with their interaction and relative pathways, it can indicate the aberrant physiological conditions or developmental stages of the samples.

In this study, we employed microarray-based approach to investigate the miRNA expression profiles of YQFM in treating chronic heart failure and found out pivotal differentially expressed miRNAs. By further predicting related gene targets and pathways, we found the anti-CHF effects of YQFM may be attributed to preventing cardiac hypertrophy and apoptosis, which were further validated by *in vitro* cellular models. This systematic approach to investigate the miRNA expression profiles and pharmacological validation of Yiqifumai Injection improved the understanding of mechanism of action of Shengmaifang, and provided the basis to its effective components discovery.

## Materials and methods

### Animal experiments

In this study, all procedures that involved animals were performed in accordance with institutional guidelines and with approval from the Animal Use Committee of Tianjin University of Traditional Chinese Medicine. 30 male wistar rats (180~200 g) were purchased from Beijing Vital River Laboratory Animal Technology Co., Ltd. (Beijing, China). All rats were reared under standard laboratory conditions. A chronic heart failure (CHF) model was produced secondary to ligation of the left anterior descending coronary artery. Briefly, rats were anesthetized by 5% chloral hydrate. A left thoracotomy was performed at the 5th intercostal space, the heart was rapidly exteriorized, and the left anterior descending coronary artery was ligated by a 6~0 polypropylene suture, placed approximately 2–3 mm distant from its origin, and tied securely. Subsequently, the heart was returned to its original position and the incision was closed. 400,000 U penicillin per rat was injected after surgical treatment.

If ST segment elevated more than 0.2 mV on electrocardiograms and echocardiography was used to detect ejection fraction (EF) values were between 38% and 50% after 24 h surgical treatment, it was suggestive of myocardial infarction (MI). Sham-operated rats similarly treated without ligature served as controls. The surviving rats (20 MI rats and 10 Sham rats) were housed and maintained on standard chow. All procedures involving animals and their care in this investigation conform to the Guide for the Care and Use of Laboratory Animals published by the US National Institutes of Health (NIH Publication No. 85–23, revised 1996). The rats were maintained for a period of 4 weeks and alive ones were randomly separated into three groups (*n* = 10) as follows: Sham-operated group (Sham, physiological saline, i.p.), Myocardial Injury group (MI, physiological saline, i.p.) and Yiqifumai Injection-treated group (YQFM, 5 mg/Kg/d, i.p.). Drug treated rats were administrated for 28 days, Sham and MI rats received an equivalent volume of physiological saline each day.

### Echocardiography detection and left ventricular intubation evaluation

Echocardiography was used to detect ejection fraction (EF), fraction shortening (FS) and peak velocity (Peak Vel) by Vevo2100 Ultrasonic doppler instrument (VisualSonics, Canada) at 4 weeks after the operation as performed in our previous study. Left ventricular intubation (Millar, USA) evaluated maximum rate of left ventricular systolic pressure rise (dP/dt max), cardiac output (CO) and stroke work (SW). BNP and c-TNT levels were evaluated with an enzyme linked immunosorbent assay (ELISA) kit according to the manufacturer's instructions.

### Histopathology and immunohistochemical examination

Rats underwent a left thoracotomy under anesthesia (chloral hydrate i.p.), and heart sections were fixed with 4% paraformaldehyde (PFA) and treated with masson-trichrome staining for histopathological examination. Heart sections was made into paraffin sections. After general dewaxing and antigen reparation, the tissue slices were overlayed with primary antibodies, including B-cell lymphoma-2 (Bcl-2, Proteintech, China) and B-cell associated X (BAX, Proteintech, China) at 4°C overnight. Slides were incubated with the biotinylated secondary antibodies for 30 min at 4°C and then with a streptavidin (Dako, Kyoto, Japan). Color development was accomplished using DAB as a chromogen.

### Agilent rat miRNA microarray experiment and data analysis

Total miRNAs were extracted from tissues in the left ventricle near infarction area of rats, using mirVanaTM RNA Isolation Kit according to the manufacturer's specifications. Total miRNA was quantified by the NanoDrop ND-2000 (Thermo Scientific) and the miRNA integrity was assessed using Agilent Bioanalyzer 2100 (Agilent Technologies). The sample labeling, microarray hybridization and washing were performed based on the manufacturer's standard protocols. Briefly, total miRNA was dephosphorylated, denaturated and then labeled with Cyanine-3-CTP. After purification the labeled miRNAs were hybridized onto the microarray. After washing, the arrays were scanned with the Agilent Scanner G2505C (Agilent Technologies).

Feature Extraction software (version10.7.1.1, Agilent Technologies) was used to analyze array images to get raw data. Next, Genespring software (version 12.5; Agilent Technologies) was employed to finish the basic analysis with the raw data. Both raw data and normalized data have uploaded into GEO database as the accession number of: GSE106547. Differentially expressed miRNAs were then identified through fold change (fc) and Reverse Rate (RR). The threshold set for up- and down-regulated genes was a fold change ≥4 (or ≤1/4) and a Reverse Rate (RR) from 1 to 2. Hierarchical Clustering was performed to show the distinguishable miRNAs expression pattern among samples. Target genes of differentially expressed miRNAs were the intersection of the prediction by 2 databases: Diana microT-CDS v5.0 (Reczko et al., 2012; Paraskevopoulou et al., 2013) and miRwalk v3.0 (Dweep et al., 2011; Dweep and Gretz, 2015). DAVID Bioinformatics Resources v6.8 was used to determine the function of target genes and predicted signaling pathways. String v10.5 and Cytoscape v3.3.0 was employed to construct the network of target genes.

### Real-time PCR of differential miRNAs

Quantification of miRNAs was performed with a two-step reaction process: reverse transcription (RT) and PCR. Each RT reaction consisted of 1 μg RNA, 4 μl of miScript HiSpec Buffer, 2 μl of Nucleics Mix and 2 μl of miScript Reverse Transcriptase Mix (Qiagen, Germany), in a total volume of 20 μl. Reactions were performed for 60 min at 37°C, followed by heat inactivation of RT for 5 min at 95°C. The 20 μl RT reaction mix was then diluted × 5 in nuclease-free water and held at −20°C. Real-time PCR was performed with 10 μl PCR reaction mixture that included 1μl of cDNA, 5 μl of 2 × LightCycler® 480 SYBR Green I Master (Roche, Swiss), 0.2 μl of universal primer (Qiagen, Germany), 0.2 μl of microRNA-specific primer and 3.6 μl of nuclease-free water. Reactions were incubated in a 384-well optical plate at 95°C for 10 min, followed by 40 cycles of 95°C for 10 s, 60°C for 30 s. The microRNA-specific primer sequences were as follows: rno-miR-21-3p: CAA CAG CAG TCG ATG GGC T; rno-miR-542-3p: TGT GAC AGA TTG ATA ACT GAA A. The expression levels of microRNAs were normalized to rno-miR-92a-3p, and they were calculated by 2^−ΔΔCT^ method.

### Cell culture

Rat H9c2 myocardial cells (from rat cardiac) (Cell bank of Chinese Science Academy, Shanghai, China) were cultured in DMEM (Corning, USA) containing 10% fetal bovine serum (Sigma, USA), 100 U/mL penicillin, and 100 μg/mL streptomycin (Gibco, USA). All the cells were grown in 5% humidified CO_2_ atmosphere at 37°C.

### Myocardial hypertrophy cell model and measurement of cell surface area

H9c2 myocardial cells were grown in 96-wells and pre-incubated with angiotensin II (AngII) (0.1 μM) and YQFM (2.5 mg/ml) for 48 h. After Fixing cells by 4% formaldehyde and increasing cellular permeability by 0.1% Triton X-100, cell fluorescence was performed by incubating for 15 min with Alexa Fluor® 488 Phalloidin (1:20, CST), to stain the F-actin, and Hoechst 33342 (1:1,000, Sigma), to stain the nucleus. After being washed with PBS, cell fluorescence was visualized using Leica DMI 6000 Inversed Fluorescent Microscope (Leica, Germany). Photographic images were collected from randomly selected fields. Cardiomyocytes surface area was determined from four pictures each group using Image-Pro Plus 6.0 by total cell area divided total cell number.

### Real-time PCR of mRNA

Relative mRNA expression of *ANP*, a myocardial hypertrophic marker was detected by Real-time PCR in AngII induced hypertrophy H9c2 myocardial cells. Total RNA was extracted using RNeasy® Mini Kit (QIAGEN, Germany) according to the manufacturer's protocol. Two micrograms of total RNA were used to synthesize cDNA with QuantiTect Rev.Transcription Kit (QIAGEN, Germany) using random primers. 50 ng of total cDNA was used for PCRs by GenElute™ QuantiFast SYBR Green PCR Kit (QIAGEN, Germany). Primers specific to the atrial natriuretic peptide (*ANP*) gene (Fw: GGG AAG TCA ACC CGT CTC A; Rev: GGC TCC AAT CCT GTC AAT CC, Sangon, China) and Rat *GAPDH* Endogenous Reference Genes Primers (Sangon, China) were used to PCR. The expression level of *ANP* was normalized to *GAPDH*, and they were calculated by 2^−ΔΔCT^ method.

### Cell apoptotic model and Hoechst 33342 staining

H9c2 myocardial cells were seeded in 96-wells at the density of 4,000/well. The cells were pre-incubated with YQFM (2.5 mg/ml) for 24 h before exposed to tert-butyl hydroperoxide (t-BHP) for 1 h. Intracellular Nuclear DNA in treated cells contained in 96-well plates was visualized by staining with the DNA-specific dye Hoechst 33342 at a final concentration of 5 mg/ml. Cells were observed immediately with filters for blue fluorescence.

### Western blot analysis

H9c2 myocardial cells were seeded in 6-wells at the density of 100,000/well. The cells were pre-incubated with YQFM (2.5 mg/ml) for 24 h before exposed to tert-butyl hydroperoxide (t-BHP) for 1 h. The expressions of p-Akt and Akt were measured by Western Blot. Briefly, H9c2 cardiomyocytes with or without oxidative stress and YQFM treatment were rinsed twice with PBS buffer and lysed with a lysis buffer for Western blot and IP on ice to extract the protein. After quantification of protein concentration by BCA protein assay (Thermo Scientific, USA). The protein was separated by SDS-PAGE gel electrophoresis and transferred onto PVDF membrane. Membrane was blocked with 5% nonfat dry milk for 2 h at room temperature. Antibodies against p-Akt (Cell Signaling Technology, USA), Akt (Cell Signaling Technology, USA), HRP-labeled Goat Anti-Rabbit IgG (H+L) (Beyotime, USA) and GAPDH (Hangzhou HuaAn Biotechnology Co., Ltd, China) were used at 1:1,000. The antigen-antibody complexes were then detected with an ultra-sensitive-enhanced chemiluminescent (ECL) substrate (Invitrogen) then visualized by GelDoc XR System (BIO RAD). GAPDH was used as internal standards.

### Measurement of ATP content and cell viability

After YQFM and t-BHP treatment, intracellular ATP content was measured by CellTiter-Glo® Luminescent Assay kit (Promega) according to the instruction of manufacture. Cell viability was measured by methyl thiazolyl tetrazolium (MTT) assay and the absorbance was recorded by a TECAN infinite F200 Multi-function microplate with wavelength 580 nm. The changes of ATP content and cell viability were calculated by comparing the luminescent or absorbance signal of the treated cells with that of untreated H9c2 myocardial cells.

### Statistical analysis

All values were expressed as the means ± SD. Unpaired Student's t-test and One-Way ANOVA were used to analyze differences among groups. Statistical analysis was performed using GraphPad Prism. *P*-values of less than 0.05 were considered statistically significant.

## Results

### YQFM improved cardiac functions of rats with chronic heart failure

**Figure 1:**
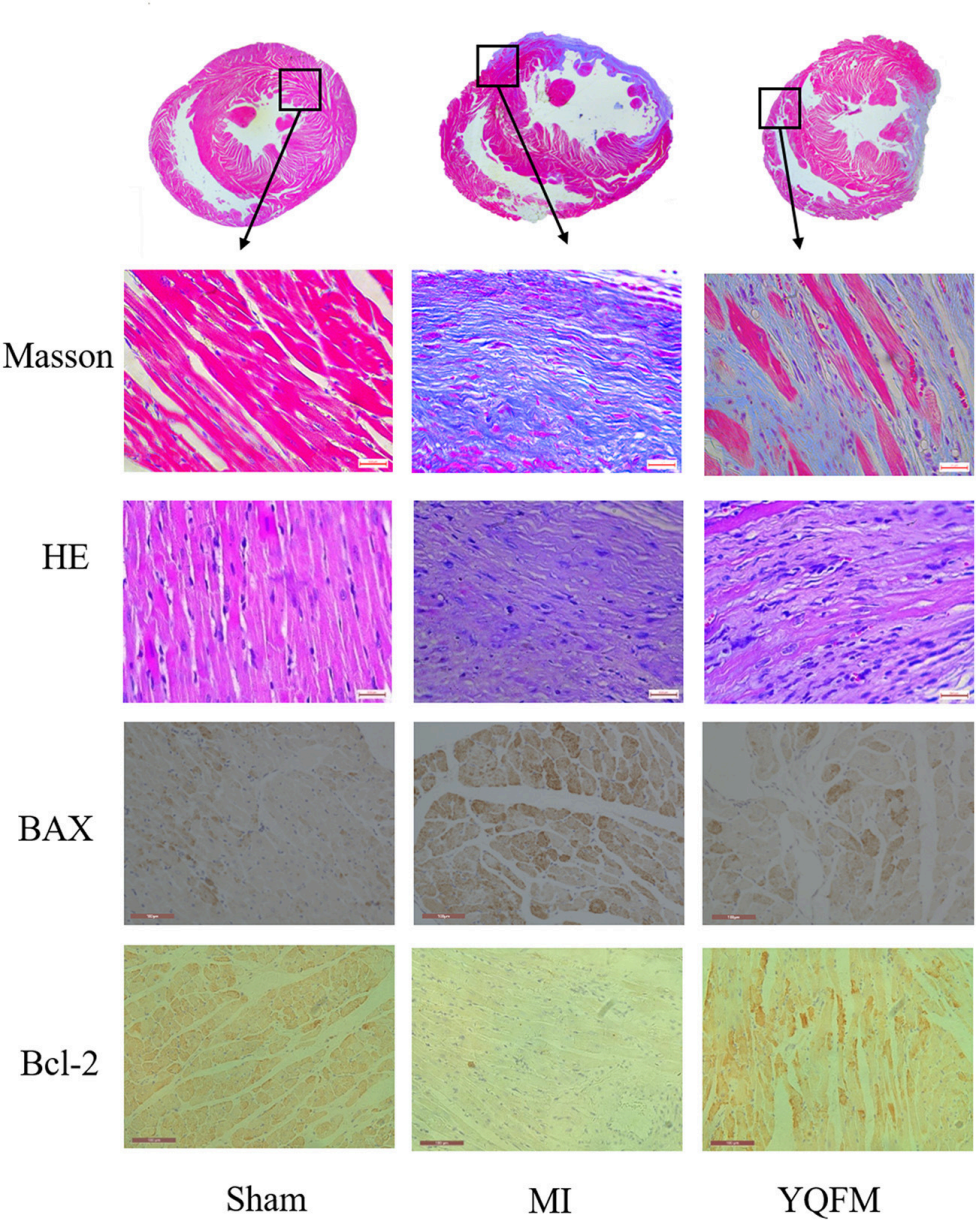
Cardiac function evaluation with histopathology and immunohistochemical examination. Masson's trichrome and HE staining image (value of scale bar is 640 μm); Immunohistochemical image of BAX and Bcl-2 (value of scale bar is 100 μm).

Rats with extensive myocardial infarction were regarded as a reliable model for chronic heart failure. As shown in Figure 1, Masson's trichrome and HE staining images showed that the MI group existed myocardial fibrosis caused by CHF. YQFM treatment could inhibit hyperplasia of interstitial fibers in myocardium, thus reversed ventricular remodeling process. The immunohistochemical results of myocardial tissue showed that YQFM obviously reduced expression of BAX and increased expression of Bcl-2. It indicated that YQFM might reduce myocardial apoptosis through regulating expression of BAX and Bcl-2.

**Figure 2:**
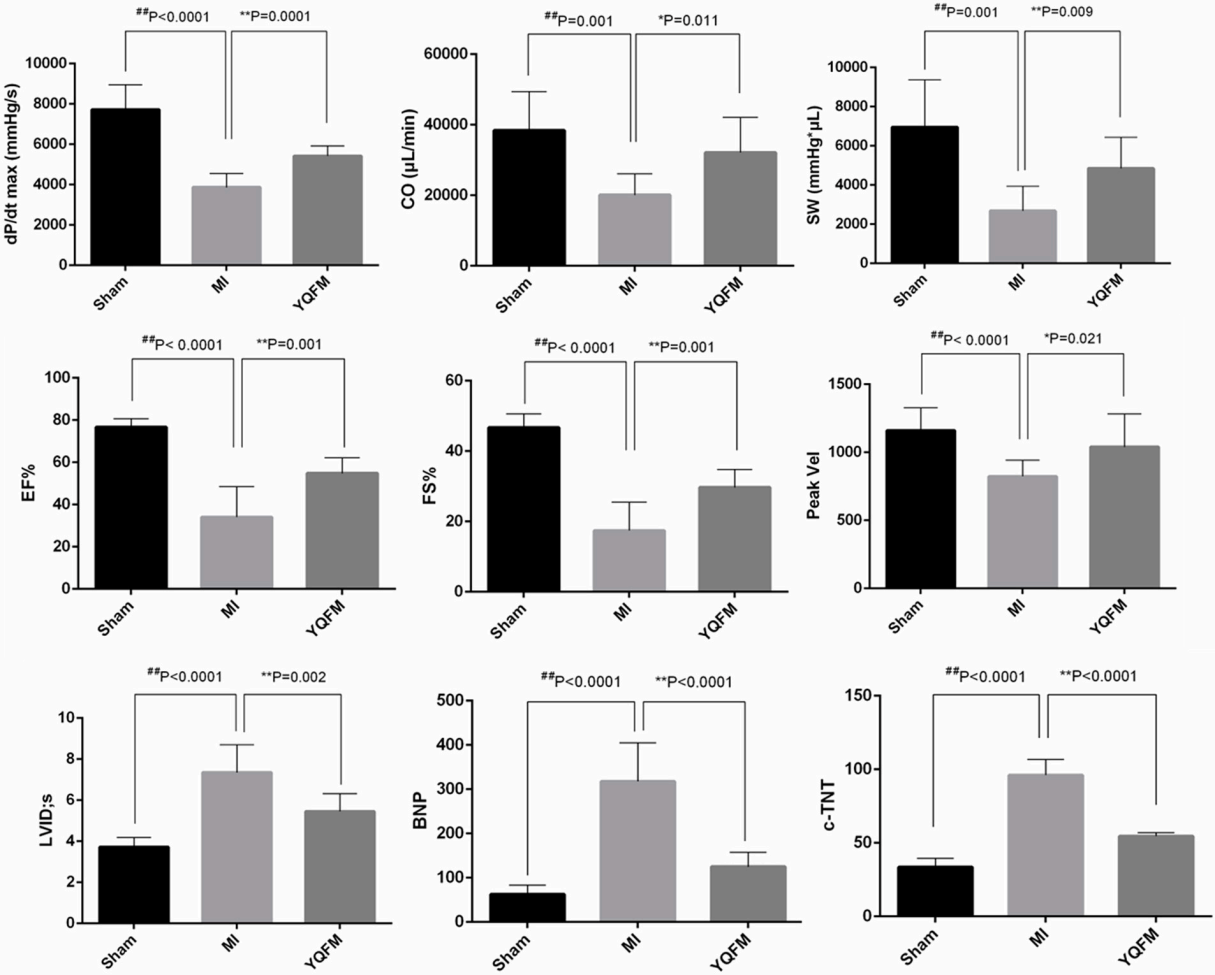
Cardiac function evaluation with echocardiography detection (*n* = 10), left ventricular intubation evaluation (*n* = 8) and immunohistochemical examination (*n* = 8). All data are shown as the mean ± S.D. Unpaired Student *t*-test was performed. ^*^P < 0.05, ^**^P < 0.01, vs. MI group; ^##^*P* < 0.01 vs. Sham group.

The result of PV-loop showed a markedly decreased maximum rate of left ventricular systolic pressure rise (dP/dt max), cardiac output (CO) and stroke work (SW) (*P* < 0.01) in MI group. However, YQFM treatment could obviously increase dP/dt max (*P* < 0.01), CO (*P* < 0.05), and SW (*P* < 0.01), indicating that YQFM could effectively improve adverse physiological function caused by CHF. The cardiac EF and FS of MI group, which reflects the overall cardiac function, were significantly decreased to 50% or less compared to Sham groups (*P* < 0.01), suggesting the cardiac function was significantly reduced. In YQFM group, EF and FS were significantly increased (*P* < 0.01), which indicated that YQFM could protect overall cardiac function against CHF. In the aspect of arterial hemodynamics, YQFM group effectively ameliorated the aortic outflow peak velocity (Peak Vel) (*P* < 0.05). The left ventricular internal diameter (LVID) in MI rats significantly expanded in the end-diastolic phase (*P* < 0.01) compared to the Sham-operated group. However, YQFM significantly reduced it (*P* < 0.01). Meanwhile, YQFM could also decrease BNP (*P* < 0.01) and c-TNT (*P* < 0.01) levels in order to protect cardiomyocytes from hypertrophy and cardiac dysfunction (Figure 2).

### MiRNAs expression profiling analysis

**Figure 3:**
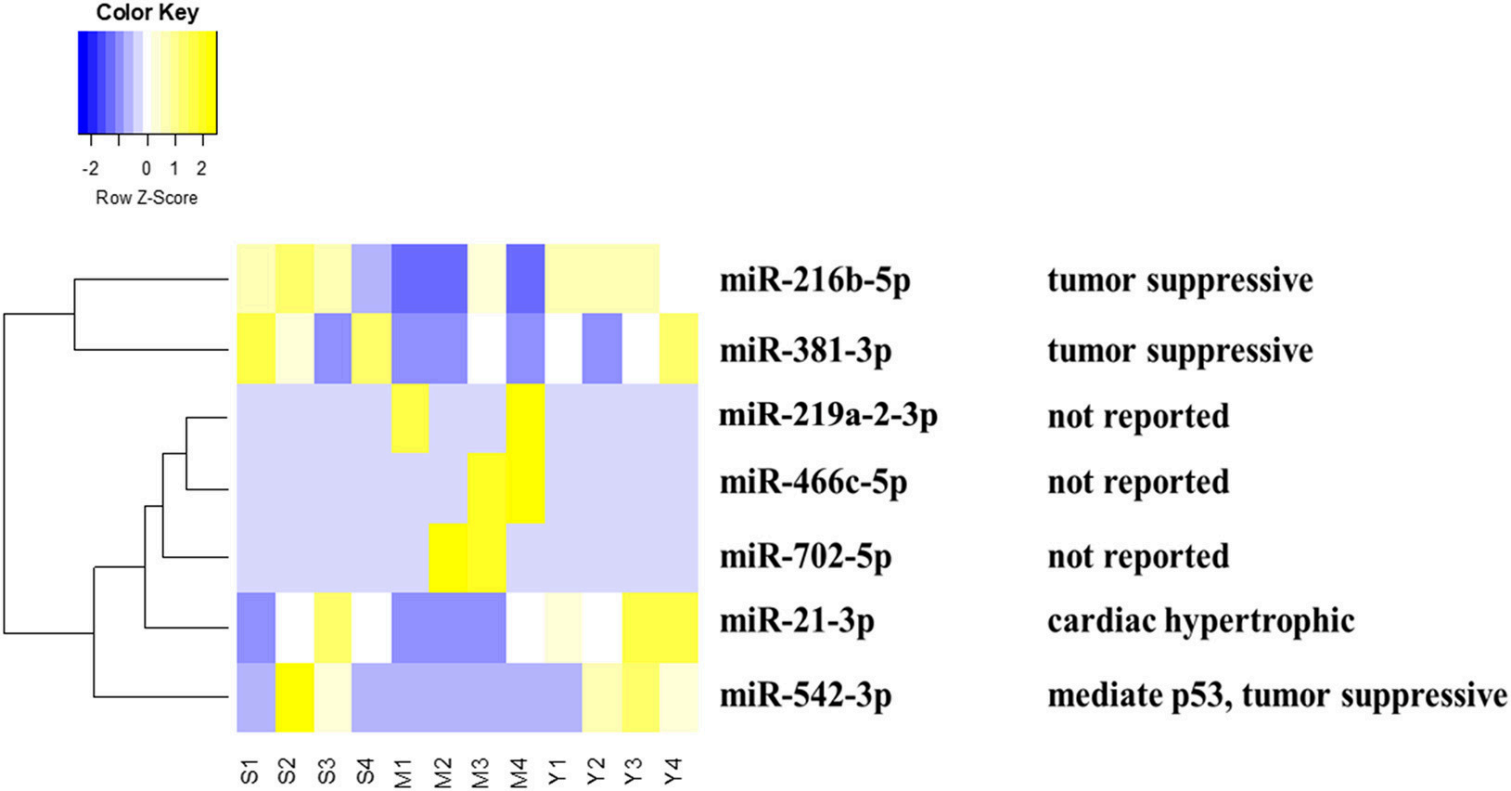
Heatmap of differential miRNAs by hierarchical clustering analysis: The S (1,2,3,4), M (1,2,3,4), Y (1,2,3,4) represents 4 samples in Sham, MI, and YQFM groups, respectively. The relative function of each miRNA was also listed.

MiRNA chip technology was used to investigate the alteration of miRNA expression profiling in the present study. We analyzed the effect of YQFM on miRNA expression in myocardial tissue of CHF rat. Finally, as Table 1 showed, seven differentially expressed miRNAs were picked out complying with fold change (fc)≥4 (or ≤1/4) (both Sham group and YQFM group compared to MI group) and Reverse Rate (RR) (YQFM group compared to MI group) between 1 and 2. Finally, miR-21-3p, miR216-5p, miR219a-2-3p, miR381-3p, miR466c-5p, miR542-3p, and miR-702-5p were considered as the differentially reversed miRNAs regulated by YQFM. These miRNAs were analyzed by hierarchical clustering, showing the main established function of each one (Figure 3).

**Table 1:** Differential miRNAs among all groups.

**Name**	**MI vs. Sham**	**YQFM vs. MI**	**fc(Sham/MI)**	**fc(YQFM/MI)**	**RR**
miR-21-3p	Down	Up	4.04	6.76	1.67
miR-216b-5p	Down	Up	4.34	4.45	1.03
miR-219a-2-3p	Up	Down	0.05	0.05	1.00
miR-381-3p	Down	Up	5.73	4.19	0.73
miR-466c-5p	Up	Down	0.05	0.05	1.00
miR-542-3p	Down	Up	38.63	42.41	1.10
miR-702-5p	Up	Down	0.05	0.05	1.00

According to recent reports, miR-21-3p could regulate cardiac hypertrophic response (Bang et al., 2014; Romay et al., 2015; Yan et al., 2015), inflammatory reaction (Yan et al., 2015), vascular reparation (Zahedi et al., 2016), aortic Stenosis (Jing et al., 2016), sepsis-associated cardiac dysfunction and cancer (Báez-Vega et al., 2016; Wang et al., 2016a). miR-216b-5p was connected with liver detoxication (Dluzen et al., 2016). miR381-3p is related to suppressive tumor proliferation (Xiao et al., 2017). miR-542-3p could modulate expression level of p53 (Wang et al., 2014), cell apoptosis and was connected with cancer (Wang et al., 2016c), ischemic stroke (He et al., 2016), liver failure (Ding et al., 2015), neointimal formation (Qian et al., 2015), diabetic cardiomyopathy and osteogenesis (Chavali et al., 2014; Kureel et al., 2014). miR219a-2-3p, miR466c-5p, and miR-702-5p were rarely reported for their functions so far.

Since miR-21-3p is related to myocardial hypertrophy and miR-542-3p is regulated to p53 expression and more importantly, both of them were regulated by YQFM, we hypothesized that YQFM might have the effects against cardiac hypertrophy and apoptosis.

### Target gene prediction and pathway analysis

Two databases were used in predicting target genes of differential miRNAs. Finally, 1473 and 5732 target genes gained from each software. Taking intersection of predicted genes, 744 common genes were generated for further analysis.

**Figure 4:**
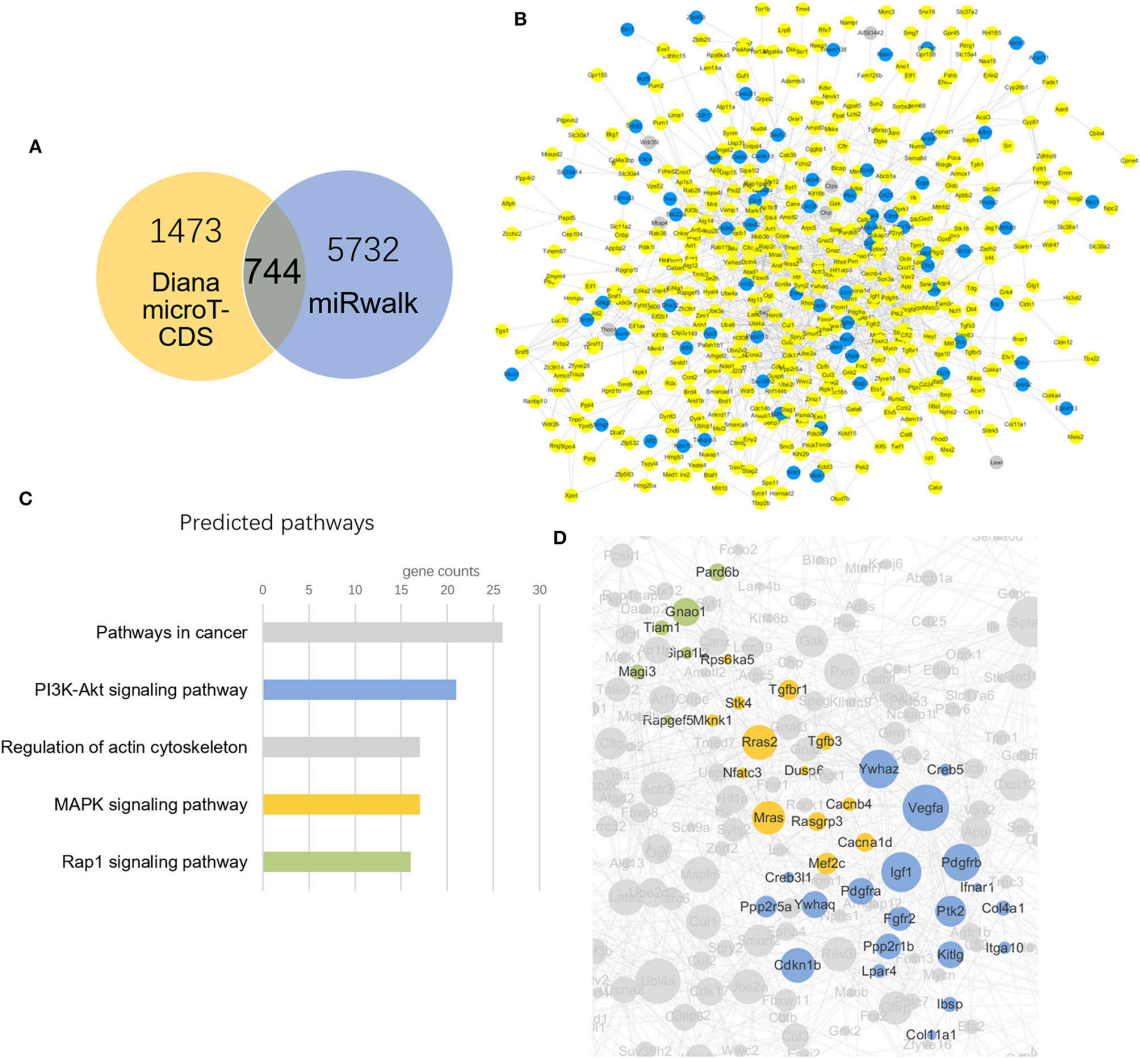
Target genes prediction and network construction. **(A)** Venn diagram of target gene counts from two different databases, taking the intersection of them finally got 744 miRNAs for further study. **(B)** YQFM Regulated target genes according to differential altered miRNAs: (yellow: up-regulated, blue: down-regulated, gray: not regulated); **(C)** Signaling pathway prediction ranking top 5 from KEGG database. The colors show consistent with that in image **(D)**; **(D)** Genes in 3 predicted pathways clustering in the network according to KEGG database.

The 744 target genes were put into String software in order to obtain interaction network. The up-regulated target genes were colored yellow and down-regulated were in blue according to the regulation status of differentially altered miRNAs (Figure 4A). There were 167 target genes up-regulated (yellow) and 30 down-regulated (blue) mediated by YQFM (Figure 4B). The target genes were imported into KEGG database and we chose top 5 pathways according to a descending order of gene counts as the important ones (Figure 4C). The results showed that PI3K-Akt and MAPK signaling pathways in the top 5 pathways were related to cardiomyocyte hypertrophy and apoptosis, indicating that YQFM might attenuate cardiomyocyte hypertrophy and apoptosis through PI3K-Akt and MAPK signaling pathways. The involved genes were highlighted in partial gene network with different colors. It also showed a positive correlation between the importance of genes and node size (Figure 4D).

### Verification of miRNAs

**Figure 5:**
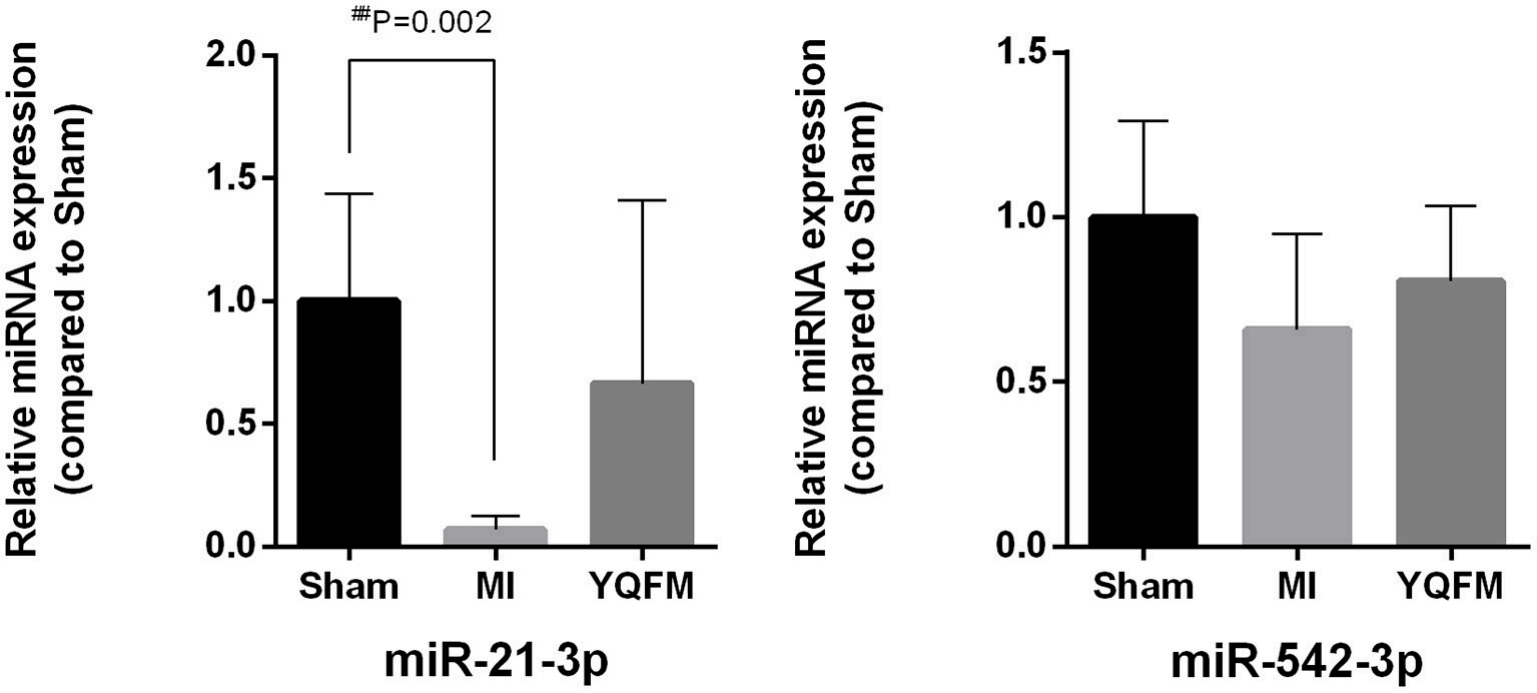
MiR-21-3p and miR-542-3p expression for validation of miRNA chip (*n* = 5). Unpaired Student *t*-test was performed. ^*##*^*P* < 0.01 vs. Sham group.

MiR-21-3p and miR-542-3 were further verified by RT-PCR. The result clearly indicated that the expression of miR-21-3p significantly decreased in MI group compared to sham-operated controls. However, the *p*-values of miR-21-3p and miR-542-3p between YQFM treated and MI group are 0.11 and 0.40, respectively, which is not statistical significant. It may be attributed to the individual differences of five biological replicates. Although there is no significantly reversed, it still exhibited obvious reversed expression of two miRNAs after YQFM treatment (Figure 5). It showed the same variation trend as the result of miRNA chip. The verification result further suggested YQFM has the effect against cardiomyocyte hypertrophy and apoptosis by regulating related miRNAs.

### Validation of YQFM against myocardial hypertrophy

**Figure 6:**
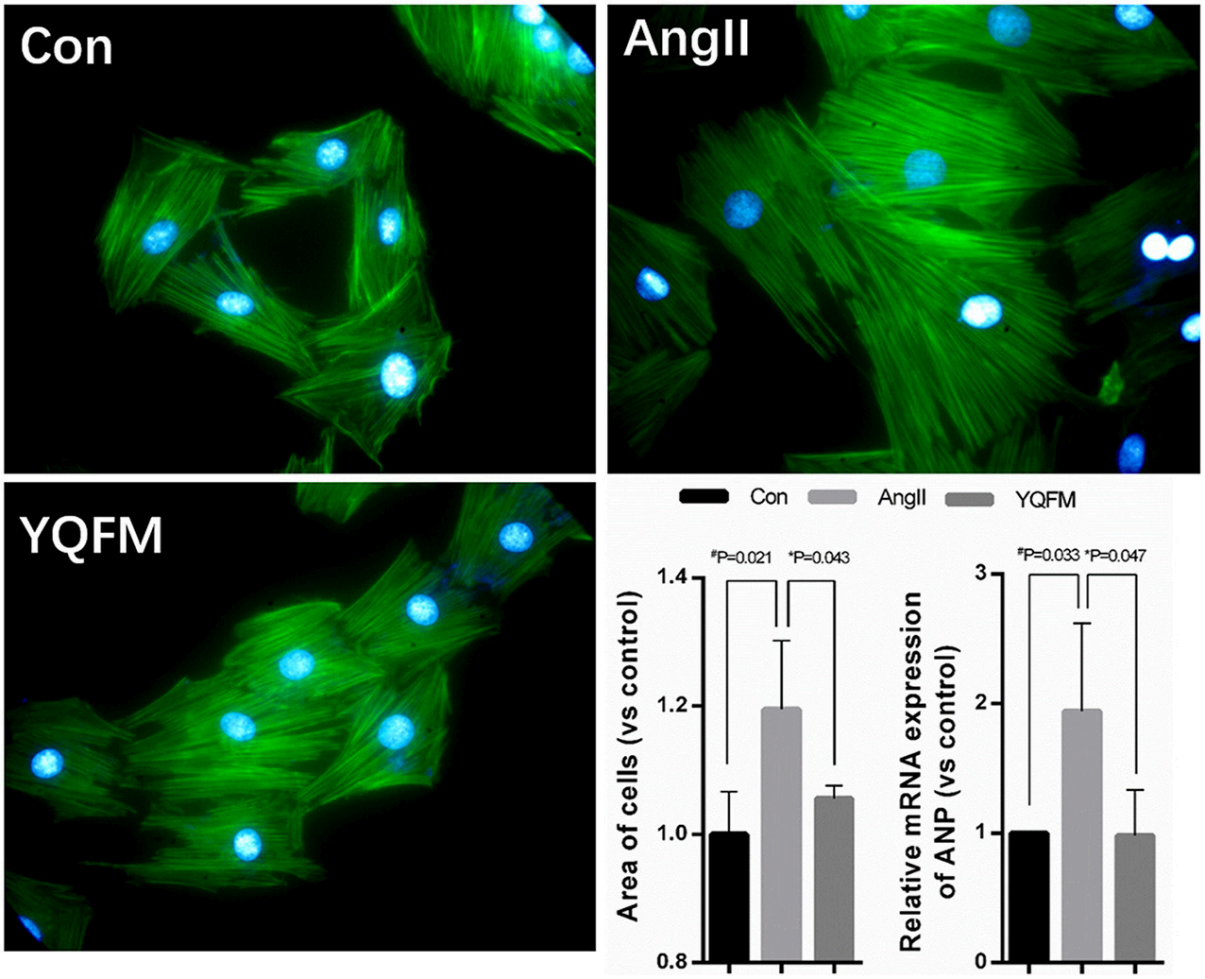
Changes of hypertrophy cell areas and *ANP* expression level (*n* = 4). One-way ANOVA was performed. ^*^P < 0.05, vs. AngII group; ^#^*P* < 0.05, vs. Control group.

Myocardial hypertrophy is a powerful compensatory state of cardiac muscle cells. Chronic heart failure develops gradually based on the myocardial compensatory hypertrophy. Thus, we used angiotensin II(AngII)to induce hypotrophy of H9c2 myocardial cells, and applied Phalloidin to stain F-actin and Hoechst to stain the nucleus. As Figure 6 showed, AngII induced larger cell surface area (*P* < 0.05), indicating a hypertrophic phenomenon of H9c2 myocardial cells. However, YQFM treatment (*P* < 0.05) significantly attenuated the surface area of cells. By estimating hypertrophic marker gene *ANP*, YQFM treatment showed a significant decrease of *ANP* expression than AngII-induced hypertropic H9c2 myocardial cells.

### Validation of YQFM against myocardial apoptosis

**Figure 7:**
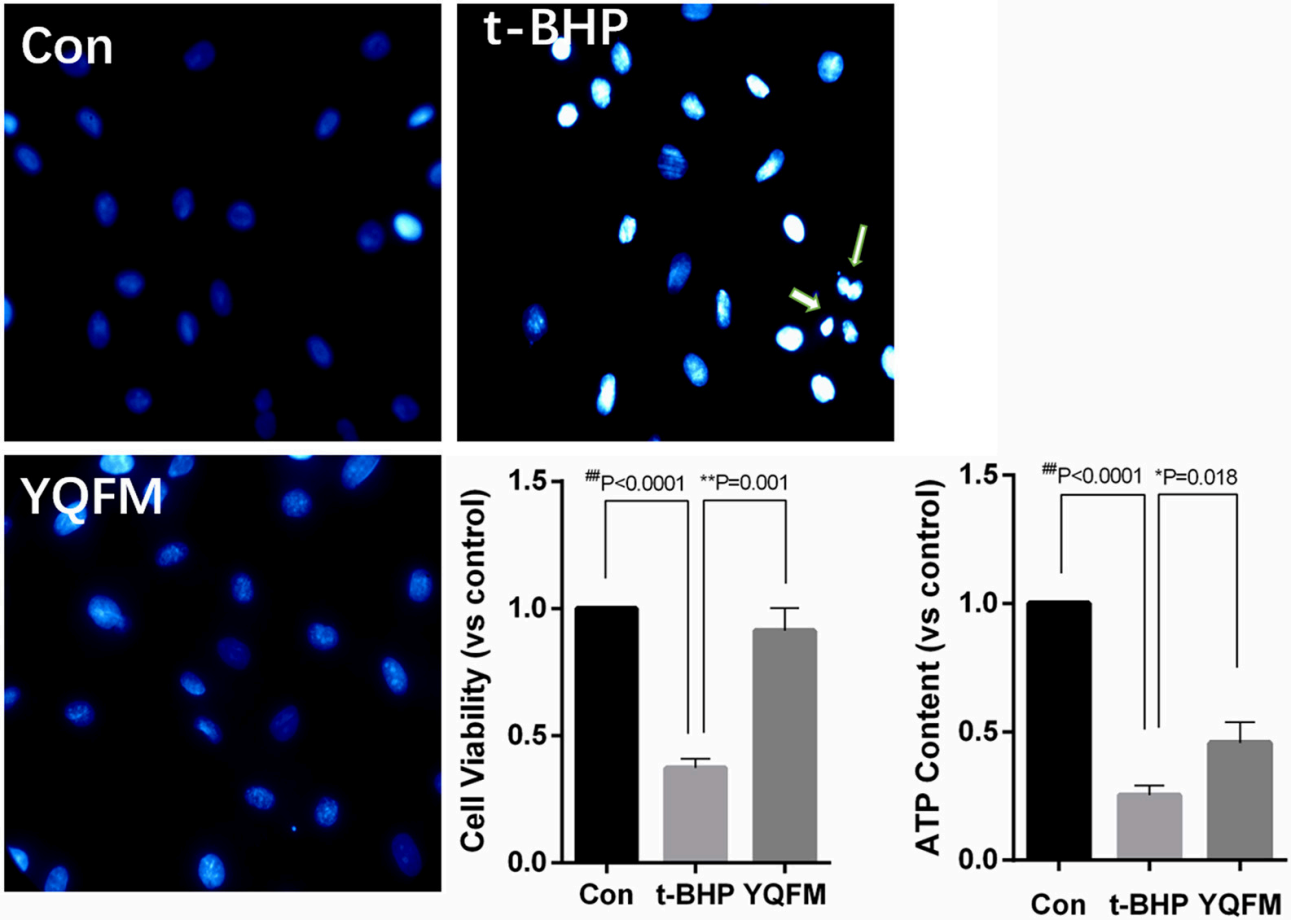
Hoechst staining, cell viability and ATP content of t-BHP injured H9c2 myocardial cells (*n* = 3). One-way ANOVA was performed. ^*^P < 0.05, ^**^P < 0.01, vs. t-BHP group; ^*##*^*P* < 0.01 vs. Control group.

After YQFM pre-protection, H9c2 myocardial cells were treated with 300 μM tert-butyl hydroperoxide (t-BHP) for 1 h, Hoechst 33342 was used to examine cell apoptotic effect. The results showed that the nucleus was dyed into distinct bright blue and fragmentation of nucleus appeared after t-BHP treatment. Nucleus of cells pre-protection with YQFM was dye into ordinary blue without fragmentation (Figure 7). Besides, YQFM might significantly improve cell viability (*P* < 0.01) which was decreased by t-BHP (*P* < 0.01). ATP content examination showed that ATP level of t-BHP injured cells was decreased (*P* < 0.01), and YQFM attenuated this effect (*P* < 0.05). Thus YQFM had the effect of anti-apoptosis on t-BHP induced H9c2 myocardial cells apoptosis model.

**Figure 8:**
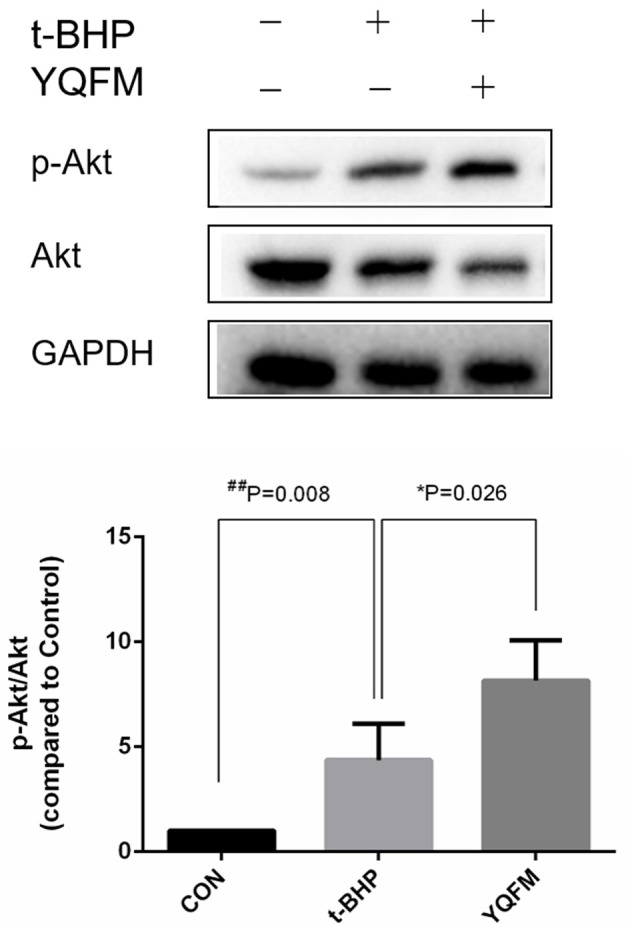
Involvement Akt expression by YQFM against myocardial apoptosis. Densitometry analysis showed the protein expression of p-AKT and AKT. Unpaired Student *t*-test was performed (*n* = 4). ^*^P < 0.05, vs. t-BHP group; ^*##*^*P* < 0.01 vs. Control group.

According to the network analysis in the previous result. YQFM has the potential in regulating PI3K-Akt pathway against myocardial apoptosis. Therefore, the protein expression of p-Akt/Akt in t-BHP injured H9c2 myocytes after YQFM treatment was measured. As shown in Figure 8, YQFM significantly elevated the phosphorylation of Akt compared to untreated cells, suggested that activation of Akt may be attributed to anti-apoptosis effect of YQFM. Our findings are accordance with the previous reports (Cui et al., 2013; Liu et al., 2016).

## Discussion and conclusion

Yiqifumai Injection (YQFM) is widely used for treating microcirculatory disturbance-related diseases in China, and was approved by China Food and Drug Administration (Yuan et al., 2011). Currently, most of the pharmacological studies of YQFM demonstrated that it has antioxidant, anti-inflammatory and other protective effects (Xing et al., 2013). To uncover the mechanism of YQFM in a holistic way, our present study used miRNA expression profiling to illustrate the effects of YQFM on CHF-rats.

By employing the echocardiography detection and left ventricular intubation evaluation, YQFM showed a marked improvement of cardiac function of CHF-rats. According to histopathology and immunohistochemical examinations, YQFM inhibited hyperplasia of interstitial fibers in order to improve cardiac function. These results consolidated that the altered miRNAs regulated by YQFM on CHF-rats were the representative miRNAs that might have the potential to explain YQFM mechanism in the further miRNA expression profiling study.

Considering both fold change (fc) and reverse rate (RR) of miRNA expression analysis are important, we utilized a strict criteria of fc≥4 (or ≤1/4) and <1 RR <2 to pick out altered miRNAs (Li et al., 2014). Finally, 7 differential miRNAs were picked out. Among them, miR-21-3p, miR216-5p, and miR542-3p are related to myocardial hypertrophy, cancer and apoptosis, respectively. The close relationship between these miRNAs especially for miR-21-3p and miR-542 and myocardial function drew our attention. MiR-21-3p has been reported to ameliorate cardiac hypertrophy respond (Bang et al., 2014). MiR-542 has been reported to inhibit the cancer cells from proliferation, invasion and migration (Cai et al., 2015; Yuan et al., 2017). By regulating them, YQFM might protect cardiomyocytes from hypertrophy and aberrant cell cycle.

To further depict and study the miRNA function, we predicted the target genes of all seven miRNAs and constructed gene network and pathway enrichment. The so-called 744 differentially expressed genes (predicted by miRNAs) were chosen as intersection of two databases. According to the regulation status of their miRNAs, the genes altered status was depicted in the gene network. More genes were up-regulated than down-regulated by YQFM in CHF-rats. Combined with the pathway enrichment results, 3 clusters of genes indicating different pathways were highlighted prominently in the gene network. We chose the top 5 pathways according to the gene counts involved in the pathways. The results showed that PI3K-Akt signaling pathway (contained 21 genes), MAPK signaling pathway (contained 17 genes) and Rap1 signaling pathway (contained 16 genes) indicated that the myocardial protective effect of YQFM. Among them, PI3K-Akt signaling pathway and MAPK signaling pathways are two notable pathways related to cardiomyocyte hypertrophy and apoptosis (Song et al., 2015; Sun et al., 2016; Xia et al., 2016). In the earlier report, Yu and colleagues found YQFM Powder Injection could attenuate myocardial remodeling and heart failure through modulating MAPKs signaling pathway, which is further supported by our findings (Pang et al., 2017).

**Figure 9:**
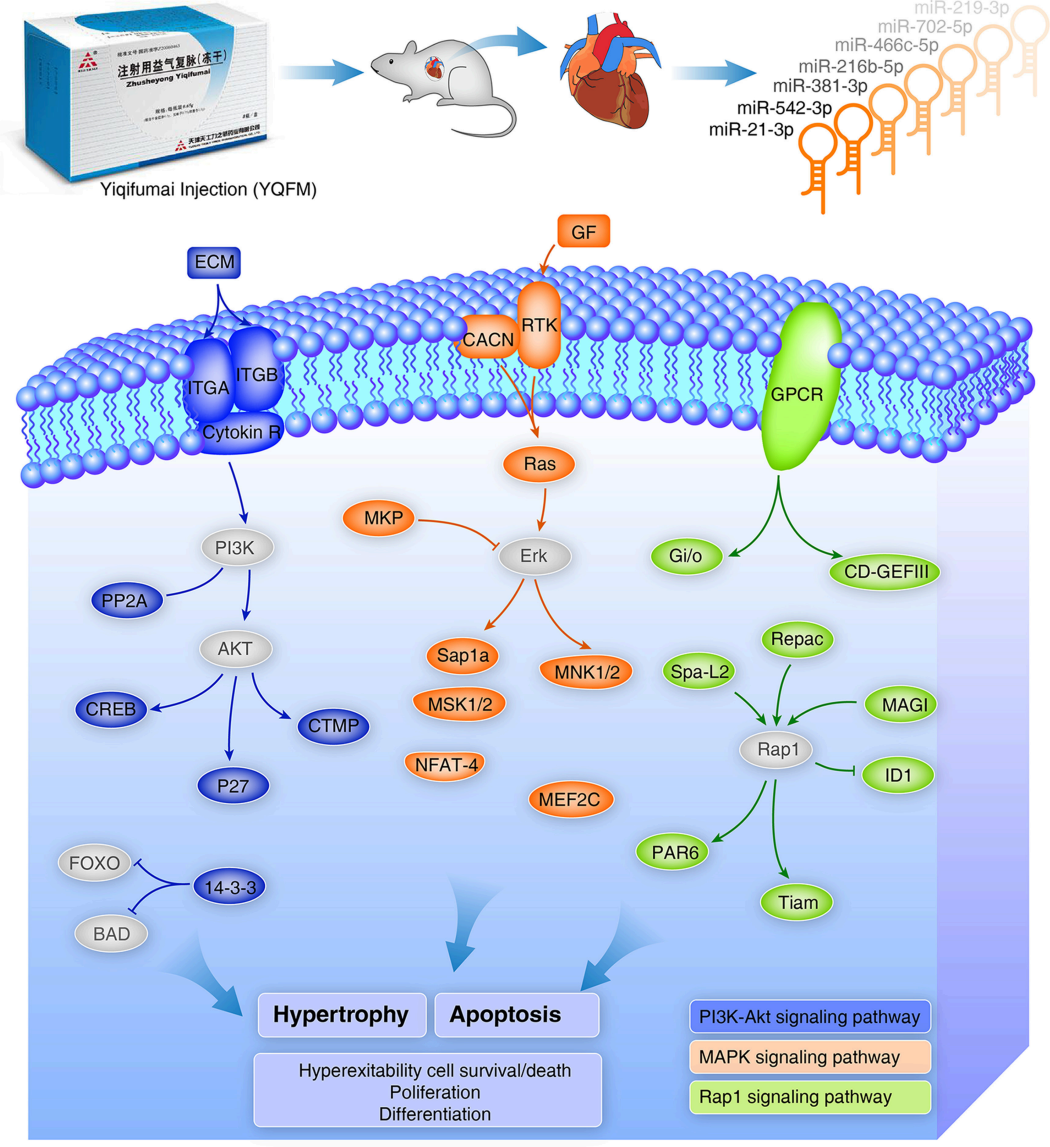
Schema of YQFM potential protective effects against hypertrophy and apoptosis by regulating miRNAs and related target genes. Colored gene icons represent genes directly mediated by YQFM in the miRNA profiling study and the key genes which were not showed up in the miRNA profiling study were added for the integrity of the figure. The blue, orange and green colors are represented for three predicted pathways: PI3K-Akt, MAPK, and Rap1 pathways, respectively. These pathways are related to Hypertrophy and Apoptosis which has been further validated on cellular model in the study. They are also related to cell survival/death, proliferation and differentiation.

Based on these findings, we presumed that YQFM could alleviate hypertrophy and apoptosis in heart tissue during CHF in order to maintain heart function by regulating the miRNAs expression such as miR-21-3p and miR-542-3p. Combined with miRNA expression profile and other results in cellular model, we speculate the genes and signaling pathways mediated by YQFM on CHF rats (Figure 9). Based on the discovery from miRNA expression profiling study, we employed angiotensin II (AngII) induced hypertrophy and tert-butyl hydroperoxide- (t-BHP) injured cellular models on H9c2 myocardial cells to estimate the protective effects of YQFM. Ang-II is an well-known neurohumoral factor which regulates the progress of myocardial hypertrophy and is considered to induce hypertrophy of myocardial cells (Liu et al., 2014; Wu et al., 2014). YQFM decreased the *ANP* expression to normal value which indicated that YQFM could protect H9c2 myocardial cells from hypertrophy induced by Ang-II. t-BHP is used for inducing cell apoptosis (Wang et al., 2016b). By pre-treated with YQFM (2.5 mg/ml) for 24 h before exposed to t-BHP (300 μM) for 1 h, the results showed that YQFM had a marked protective effect against cell apoptosis. It also improved the cell viability and increased ATP level. Moreover, we found YQFM significantly improved phosphorylation of Akt, which contributes to the anti-apoptosis effect. In summary, the cellular results showed that YQFM could protect H9c2 myocardial cells against Ang-II induced hypertrophy and t-BHP induced apoptosis. These results also illustrated that the pathways enriched by altered target genes were indeed indicating YQFM effects on protecting myocardial cells.

In conclusion, our results showed that YQFM can regulate miRNA expression in protecting heart tissue. MiR-21-3p and miR-542-3p were two important altered miRNAs found in YQFM treated group according to miRNA profiling study. We also validated the protective effect of YQFM against hypertrophy and apoptosis in cellular models. Our findings provide scientific evidence for the mechanism of action of YQFM against chronic heart failure.

## Author contributions

YW, LT, YL, and GF designed the research. GF, LL, HZ contributed to animal experiments. YW, XL, and YZ contributed to the miRNA expression profile data analysis and cellular experiments. All authors contributed to the manuscript and approved the final version of the manuscript.

### Conflict of interest statement

YL and LT were employed by company Tasly Holding Group Co., Ltd. Other authors declare no competing interests.
